# Strengthening Social Interactions and Constructing New Oral Health and Health Knowledge: The Co-design, Implementation and Evaluation of A Pedagogical Workshop Program with and for Homeless Young People

**DOI:** 10.3390/dj7010011

**Published:** 2019-02-01

**Authors:** Andrea Rodriguez, Laura Beaton, Ruth Freeman

**Affiliations:** Dental Health Services Research Unit, School of Dentistry, University of Dundee, Dundee DD1 4HN, UK; l.z.beaton@dundee.ac.uk

**Keywords:** pedagogical approaches, young people, homelessness, critical consciousness

## Abstract

Young homeless people make up nearly one-third of those experiencing homelessness. The need to provide an educative approach, to strengthen social interacting, and construct new knowledge to increase social inclusivity, is required. The aim of this qualitative exploration was to use critical consciousness as an educative tool, to co-design, implement, and evaluate a series of oral health and health pedagogical workshops to strengthen social engagement and to construct new health knowledge, with, and for, homeless young people and their service providers. An action research design permitted the simultaneous development, implementation, and evaluation of the pedagogical workshop program. A Non-Governmental Organization (NGO), providing supported accommodation for young homeless people, acted as the partner organization. Thirteen young people and five staff members from this NGO participated and co-designed eight workshops. Qualitative data collection included unstructured post-intervention interviews together with verbatim quotes from the group discussions during the workshops and from the post-workshop questionnaires. The qualitative analysis was informed by content analysis to permit the emergence of key themes from the data. The two themes were: 1. ‘trust building and collective engaging’ and 2. ‘constructing knowledge and developing skills’. Theme 1 highlighted engagement with the service provider, illustrating the transformation of the young people’s relationships, strengthening of their social interacting, and enabling their critical reflexive thinking on sensitive issues present in the homelessness trajectory. Theme 2 illustrated the young people’s ability to share, lend, and encode their new health information and convert it into an understandable and useable form. This new comprehension permitted their behavior change and social interaction. These findings provide an approach to increase young people’s knowledge, health literacy, and strengthen their social interacting to support community action.

## 1. Introduction

Recent news reports on young homeless people have put youth homelessness at center-stage. Research that was commissioned by the BBC showed that over 40% of young people sofa surfed with friends for long periods of time without seeking support from Local Authorities [1]. In Scotland, 28% of all homelessness applications were from people aged between 16 and 24 years [2]. The Scottish Government linked youth homelessness to social and health-related factors [2,3,4,5] and called for a joint and multiagency approach to tackle the health and social care challenges of young homeless people [6]. It has been shown that youth homelessness is caused by family and relationship break-down, exacerbated by youth unemployment, escalating rent costs, and overall benefit cuts [7]. The result has been poorer physical and mental health in young people experiencing homelessness [8,9]. Hence, youth homelessness has been identified as a serious and chronic social problem.

The Scottish Government developed a series of homelessness policies [10,11,12,13,14] that recognized the need for a holistic approach that not only address housing, but also other critical issues, such as physical and psychosocial needs, including the effects on mental health and oral health associated with being young and homeless. What brings all of these health aspects together is the common risk factor approach (CRFA) [15]. Oral health, in this regard, is included in the CRFA and allows dentistry and oral health promotion to be key features in addressing the social aspects of psychosocial health and wellbeing, i.e., low self-esteem, reduced employment opportunities, or isolation. Despite these policy recommendations and CFRA interventions to prevent and/or solve health and oral health problems, young people experiencing homelessness continued to experience limited access and engagement with health and social care services [16,17]. Within a lexicon of mistrust, negativity, perceived stigmatization, and acknowledged awkwardness, [18,19], significant communication barriers exist between those utilizing and those providing oral health, health, and social services. In their exploration of community health workers, Gale et al. [19] posed that the communication between health workers and clients was unusual, as their interactions were content and time-limited with none of the spontaneity of social interacting. They conceptualized this type of interaction as a ‘synthetic social interaction’, and while appropriate for health workers to communicate oral health and health service information, it inadvertently promoted a more paternalistic communication style, with a top-down approach that resulted in feelings of mistrust, stigma, and/or negativity in client groups. This work [19] questioned the approaches used and called for new creative and participative methods to engage with young people experiencing homelessness. There was a need to promote a more spontaneous and social interaction style that would permit the promotion of joint working and engagement between client and practitioner, ultimately to improve health, oral health, and psychosocial wellbeing. 

Using an educational tool to facilitate this interaction would allow engagement and effective communication between participants. Thinking in this way, the issue of young people’s apparent absence of motivation to engage with dental and health and social service practitioners, required reassessment. Rather, it was the intervention content, sometimes subject to a prejudicial judgment, together with a paternalistic style of interacting, which had the potential to exacerbate young people’s fears of stigmatization and rejection. This was observed as a disinclination to engage with health and social care professionals. The need to provide an environment to enable collaborative working for increased wider health knowledge and service engagement appeared to be urgently required.

In order to achieve engagement with services providers, critical reflexion on aspects of their reality and spontaneous interaction would be necessary to work in a cooperative manner. For Wolfe et al. [20], working cooperatively means improved psychosocial and cognitive skills, which in turn increase health learning capacity: for Freeman [21], it is how the participants encode the received information, how they make the information their own, which paves the way for better health literacy: for Freire [22], it is the practice of dialogue that helps to form critical consciousness and critical attitudes for learning, critical thinking, and action, with the formation of new knowledge. The practice of dialogue on sensitive issues, between service-user and service provider, is thus central for the development of understandable forms of health information and new life choices. The dialogue in Freire’s perspective is not just about expressing ideas from one individual to another, nor is it simply about exchanging ideas with others. The dialogue in Freire’s perspective [23] is an act of existential creation. Adopting this co-design strategy, based on Freire’s theoretical approach to nurture social interacting, would promote joint decision-making, strengthening social change, health literacy, and health information gain [15]. This would permit the health cognitions, health literacy, and numeracy needs of the young people experiencing homelessness to be acknowledged and managed. 

In essence, what is suggested is a participatory approach that is underpinned by Freire’s formulation [22,23] to increase critical consciousness about how current problems in society are perceived, to provoke critical attitudes on challenges that affect vulnerable and marginalized groups, and to promote communication for health learning capacity. Freire’s approach analyses degrees of understanding of reality and its relation to socio-cultural conditioning. The critical consciousness is characterized by the depth with which it interprets current problems, characterized by the autonomous and committed thinking that leads to socio-political engagement. Freire’s dialogic knowledge exchange procedure allows for the development of activities to encourage and support participants to bring their own knowledge and share their life experiences in order to make conscious choices of action. In this study, the workshop program on health promotion acted as a vehicle for this. 

The importance of working in groups to debate and to increase awareness of current problems in the wider context, as highlighted by Freire [23], has been emphasized by Candau & Sacavino [24] through their pedagogical workshop framework. Their framework adopts principles of Freire’s theoretical approach understanding this resource (in the form of workshops) as a tool to overcome feelings of passivity and powerlessness in the face of social problems that are experienced by vulnerable and marginalized groups. These authors, in alignment with Freire, state that it is often in the interaction with peers that identities, critical reflexion, and knowledge are strengthened and can be structured in actions that aim for transformation [24]. It would seem that the dialogue approach offered by the workshop program would improve understanding of the links between health and homelessness journeys, health literacy, and the quality of interactions and engagement between excluded young people and health and social care practitioners. On this basis, will the use of pedagogical workshops act to stimulate reflection towards a conscious practice, for the development of the critical spirit, inciting the recognition of its individual and collective story, having, as a consequence, the perspective of changing structures that generates abrupt social inequalities?

Rodriguez’s previous research with vulnerable youths in Brazil was also built around this framework to conduct community-based interventions, including their development and implementation simultaneously [25,26]. Following Freire and Rodriguez, this study adopted the view that there must be a deep understanding of the young persons’ life experiences, as a foundation to develop trust, enables engagement, mutual learning, and knowledge construction. For Rodriguez the core element of enabling engagement and strengthening of social change for excluded youths is the ability of the health and/or social care professional to respond sensitively to ‘the vulnerable young person’s health and psychosocial needs in the face of marginalization and exclusion’ [25]. In order to promote sensitivity and inclusion, it is necessary to have a forum that enables a person-centered approach and thereby shifts communication from synthetic to spontaneous social interacting, to support a process of building trust, new knowledge, the exploration of life experiences, and current life circumstances. 

We argue that co-designed interventions that adopt the above strategy [22,24], together with Rodriguez’s formulation [25,26] of shared working to strengthen social change, will ensure that their oral health and health issues will be sensitively explored. A two-pronged approach of (i) an understanding of communication and health interventions that divide, stigmatize, and label youth experiencing homelessness and (ii) the reflection and the dialogue between participants, has the potential for success. Using a pedagogical workshop strategy provides a safe space; permits young and homeless people to speak of their oral health and health concerns; and, allows for a critical reflection upon their life circumstances and enables engagement with practitioners. Therefore, the aim of this qualitative exploration was to use critical consciousness [22,23] as an educative tool, to co-design, implement, and evaluate a series of oral health and health workshops to strengthen social engagement and construct new health knowledge, with, and for, young people experiencing homeless.

## 2. Materials and Methods

The method used here to promote the active participation of a Non-Governmental Organization (NGO), practitioners, and young people was the dialogical approach proposed by Freire [22].

### 2.1. Theoretical Underpinning of the Pedagogical Workshop Program

In order to achieve successful engagement, participation and co-design, it is necessary to ensure that [i] the NGOs assisting in designing and hosting the intervention; [ii] the service providers co-delivering the activities; and, [iii] service users receiving an intervention in which their views and life experiences are integrated and they can play an active role within the whole developmental process [27].

### 2.2. Design

This qualitative exploration used an action research design to permit the simultaneous development, implementation, and evaluation of the pedagogical workshop program [28]. 

### 2.3. Procedure

#### 2.3.1. Research Context

The NGO was identified for the pedagogical workshop program specifically because of the well-known work with young people aged between 16 and 24 years who were homeless. Their remit was to ‘advise, educate and support young people’ and ‘to enable them to build life skills and the resources required to make a positive and healthy transition to adulthood’ [29]. 

#### 2.3.2. Gathering the Participants: The Sample

Initially, a convenience sample of NGO managers, and their front-line staff were invited to participate. The group was recruited by the principal investigator (PI) through previous contact with the NGO partner as a first phase of the development work (Table 1). Five of the NGO’s staff members also participated in the eight workshops and evaluation (Table 1). 

A purposive and non-probability sample of young people who were living in temporary and supportive accommodations provided by the NGO was invited to participate: their recruitment was facilitated by staff members who were working directly with them. The NGO staff were informed of the voluntary nature of the young person’s participation. The invitation to take part in the study was provided initially in the form of a poster advertisement, placed in common areas of passage of young people, inviting those interested to contact staff members. Secondly, information sheets were provided to those who requested additional information. After a cooling off period of a month, the young people who had decided to participate completed the consent form with the PI. A total of 13 young people took part in the workshop program and evaluation (Table 1).

#### 2.3.3. Pedagogical Workshop Program: Phases of Development

Phase 1: A series of meetings took place between the PI and managers, staff members of the NGO, and young homeless people living in temporary accommodation, with the intention of co-designing the content and delivery of the workshops. In these three initial meetings, an identification of four main topics on health promotion to be covered through the workshops was agreed by all participants. The meetings, in phase 1, guaranteed that mutual trust was built between the PI and the participants providing the foundation for an open exploration of key health and social concerns of young people experiencing homeless. Having jointly identified and selected key health promotion topics for the first workshops, the PI engaged with one NHS Board and one NGO to ensure that the information provided at the workshops was evidence-practice based and complied with current guidance. The first package of workshops (four) was then jointly planned, delivered, and evaluated through direct observation [30] during the workshops, and with post-questionnaires given to participants and staff members. This package of workshops was delivered by the PI with the assistance of an oral health promoter from the NHS Board and a research assistant.

Phase 2: This phase adopted a similar participatory approach as in Phase 1. Following completion of the first four workshops, the NGO and young participants required one more package of workshops. Discussion groups were conducted with all participants, and in particular the young people, to inform a second phase of workshop development. Phase 2 was characterized by an open discussion on the young participants’ additional concerns and joint workshops’ planning. Four additional concerns were identified by the homeless youth and staff members. The PI, as mentioned above, repeated the process implemented in Phase 1—once all the workshops’ content had been agreed with all participants, one key NHS Board and one NGO were involved to contribute to the health-related information provided and confirmed that it was supported by recent literature. The second package of workshops (four) were planned, delivered, and evaluated using the same format as mentioned in Phase 1 (Table 1). 

Phase 3: This phase formed part of the evaluation of the pedagogical workshop program and involved semi-structured interviews with some of the participants (Table 1). 

The structure of the pedagogical workshop program is described below. It used critical consciousness as an educative tool to support awareness of self-knowledge, critical thinking, and co-construction of consensus, and a joint action plan for behavior change. 

### 2.4. Pedagogical Workshop Program Structure and Timing

The structure and format of the workshops used Candau and Sacavino’s framework [24] with some additions and adaptations to match and reflect the life experiences of the young homeless participants. A range of creative approaches to engage with the participants, and to accommodate their different learning styles, was incorporated into the workshops. Therefore, the Candau and Sacavino’s framework [24] was not only linked with the Freire’s theoretical position of ‘tuning into the other’s universe’, but it also provided a means for its operationalization in the workshop setting. The workshops were undertaken in small groups following Freire’s approach to facilitate the discussion of sensitive issues, and used blended learning strategies. Therefore, spontaneous social interactions were fostered in the workshops by promoting lively interaction, using art, drama, music, films, photographs, popular magazines, and any form of communication that would permit the participants to raise their voices and to express their views (Table 2). These tables show the content of each of the eight workshops, together with a description of their implementation. 

The workshops were held and incorporated into the routine activities that were already in place at the NGO partner and at a time suggested by the NGO managers to meet participants’ availability. The entire workshop program (development, implementation, and evaluation) ran over a 10-month period and each workshop lasted for two hours.

### 2.5. Workshop Structure

(1) Shared meal: On arrival, and for the first 30 minutes of each the workshop, the NGO provided a meal for the young people. This meal was shared between the participants and the workshop facilitators and started a process of mutual trust and interaction, as proposed by Freire [19,20]. 

(2) Introduction: Icebreaker activities were introduced to acquaint the participants with one another, to engender a relaxed atmosphere, reduce barriers, and enable spontaneous interaction and discussion. The ice-breaker activities thus provoked openness within an informal learning environment. In this way, the spontaneity of the interaction was fostered to enable critical reflection, as described by Freire. 

(3) Increasing awareness: During the workshops, the PI and the workshop facilitators captured the different concerns, knowledge, and life experiences of each participant. This was a crucial moment because what was captured from the participants’ narratives informed the content to be explored later in the workshop. Following Freire’s formulation, the participants involved were invited by the PI to share their own perceptions, knowledge, and experiences instead of receiving health advice in a more traditional format of a health promotion session. In doing this, there was a change in the perspective of learning based not only in passing or transmitting new information and knowledge, but also putting the participants at the centre of their learning process [22]. 

(4) Deepening and reflection: The shared views and experiences of young participants during the workshops was creatively combined into a cohesive whole and conveyed as ‘new information’. The presentation of the ‘new information’ allowed a shift in awareness, an appreciation that they were not alone in their experiences and furthered a deeper critical reflection proposed by the topic(s) discussed. This step in the workshop addressed Freire’s approach [23] to enable participants to explore their society and critically question key issues, identifying and making explicit their understanding of the nature of their past and present social situations to develop increased capacity for choice.

(5) Co-construction and synthesis: In order to reach a level of consensus among the participants and to enable them to take ownership of their own and ‘new information’, it was necessary for participants to continue the discursive process by facilitating the expression of their own opinions, views, and thoughts. This process enabled each aspect or point of view to be synthesized into a co-constructed knowledge base by engaging the group in diverse and creative debate and activities. This produced a new personal knowledge consensus to be translated into key information and insights for use in the future. This critical consciousness and the confrontation of their current life circumstances allowed for them to reflect on self-esteem, feelings of being stigmatized, their responsibilities, and roles to achieve social transformation [23].

(6) Group agreement and workshop close: The aim of the closure of the workshop was to invite participants to explain individually how their increased awareness, reflections, new personal knowledge, and insights, which were explored during the workshop, would be incorporated into their lives and daily routines. This encouraged the participants to translate their personal commitments into a group’s action plan to support behavior change. Therefore, this last moment of the workshop illustrates Freire’s proposition [22] that in consequence of a critical consciousness, a critical attitude can be formed toward healthier life choices. 

### 2.6. Qualitative Data and Analysis

The intervention was evaluated using a qualitative methodology. The qualitative data included, for this purpose, direct observation and recorded discussions during the workshops, post-workshop in-depth interviews, and verbatim comments from the post-workshop questionnaires. During the workshops and the post-workshop interviews, the participants were invited to speak about the workshop experience and/or anything they wished to. They could stop talking when they wanted and could bring the interview to an end when they felt it was the right time. All of the workshops and interviews data were transcribed and subjected to content analysis [31]. 

Content analysis allows the transcribed data to be explored meticulously, line-by-line to discover categories, themes, and concepts [31]. The analysis, therefore, starts with exact and thorough line-by-line coding to identify categories and themes. The coding went beyond a simple description of the data context and therefore when an interesting idea/incident was noted this was catalogued to allow for a subsequent category to emerge. After the researchers independently examined the data they met together to discuss their categories and themes. When a disagreement occurred further discussions ensured that a consensus was reached and that the data were trustworthy.

### 2.7. Ethical Considerations

Ethical approval was obtained from the Research Ethics Committee at the University of Dundee (UREC 15149). Poster information and participant information sheets were provided, and consent forms were required to be completed prior to taking part in the workshops and post-workshop interviews (see ii). All of the data were anonymized and confidentiality ensured.

## 3. Results

### Demographic Profile of the Participants

Thirteen young people (YP) participated in the workshops; eight were female and five were male. The sample was aged between 18 and 22 years. Five NGO practitioners participated in the workshops. Two were male and three were female. All of the young people (YP) and the NGO staff members (SM) contributed to the qualitative data presented.

The qualitative findings are described below. The presentation of the themes and their behavioral descriptors provide an illustration of how critical consciousness in the form of educational workshops may be developed and implemented for and with young people to promote health and psychosocial wellbeing.

Theme 1: Trust building and collective engaging

The first theme to emerge was ‘trust building and collective engaging’. People need to feel safe in order to share their views and experiences. Trust building and collective engaging was characterized by open discussion, non-judgmental attitudes from the researcher and participants, liveliness, spontaneity, hearing and ‘sharing experiences’, thinking about life and current life circumstances, and deeper discussion of sensitive issues to achieve better health and well-being. The behavioral features of trust building and collective engaging were categorized as spontaneous social interacting and context enabling. Spontaneous social interacting reflected the motivation to share experiences and feelings in a trustful environment, and it became apparent that it supported the strengthening of the relationship between the young people themselves and with the practitioners. The following comments from the post-questionnaires are illustrative: ‘What I most liked was to see the interaction between them, young people being comfortable about sharing experiences’. (SM 4); ‘the best part of the workshop was the social discussion, really good fun’ (YP 3); ‘It was an informal chat’ (YP 4); ‘I could talk about normal life, issues related to me’ (YP 5); and, ‘We need to have more spaces like this to talk about life really helpful’ (YP 7).

Context enabling described spontaneous social interaction among the young people. Direct observation, post-questionnaires, and interviews captured their perceptions about the workshops after each session. The workshops were seen as promoting an ‘informal atmosphere’, a ‘welcoming and friendly environment’, and a ‘safe space’ to share feelings, thoughts, beliefs, and experiences ‘without judgment’. A staff member highlighted that the workshops created more opportunities for young people to express their lived experiences that otherwise might not have been revealed in a one-to-one session with the NGO staff:

“A young woman who attended the workshop around mental health, she was very vocal about her own experience of how mental health has been for her. She’s not somebody that normally expresses much in a group, she’s quite a private person, so I thought it took quite a lot for her to open up, to trust, but I also appreciate the fact that she felt she was in a really good space that she could share that experience with the others and I felt that was really valuable for the rest of the group to hear that. I think this activity [the workshops] encourages people to talk about their own experiences “. (SM 1).

The quote above suggests that the participants of the workshops were able to reveal difficult situations to their peers in the context of the group, because they felt that they were in a safe space to do so and, as these participants had a positive reception, this helped them to re-signify such experiences internally in a movement to overcome them.

An integral part of ‘context enabling’ was observed as the PI’s attention to the participants’ feedback during the process of the workshop program delivery, which promoted flexibility to change and to adapt the group activities to encourage the young people to participate, as illustrated by the comment of this staff member: 

“I do want to say that I appreciate the way you [PI] tailored the sessions in response to the feedback that you got each session... and the way that you managed to structure the workshops so that there was more discussion and more interactivity”. (SM 2)

The important behavioral aspects of trust building and collective engaging were spontaneous social interacting and context enabling and it was possible to conceptualize this theme and its behavioral dimensions as providing the ambiance for critical reflexive thinking [22]. To facilitate critical reflexive thinking there must be trust between all of the participants involved (i.e., context enabling), respect for all different knowledges in place, recognition of what needs to change/be transformed and sharing of experiences (i.e., spontaneous social interacting) for the integration of previous knowledge (experience) into ‘new knowledge’ (group commitment for action). Critical reflexive thinking in the context of the workshop intervention accepted the reality of the individuals’ narratives and allowed the young people to re-evaluate their opinions, assumptions, and life expectations. It, thus, provided a path towards the recognition and acceptance for young people experiencing homelessness, from themselves and society. The significance of promoting trust for social engagement with services and within the community is apparent and reflects Islam et al.’s conceptualization of social capital [32]. In their concept of social capital, it is the need for mutually trusting networks, seen here as a consequence of the building of critical consciousness, which will provide a pathway for engagement with dental and health services, social services, and ultimately for social inclusion. In summary, trust building and social interacting appeared to transform relationships with services, strengthen social interacting, and enable critical reflexive thinking that, in turn, allowed for the subsequent production and acquisition of new knowledge and actions. 

Theme 2: Constructing knowledge and developing skills

Giroux [33], in his exposition of Freire’s critical pedagogy, makes a differentiation between gaining knowledge and the ‘mastering of specific skills’. He states that Freire’s approach is concerned with ‘imaging literacy’ and it is this ‘mode of intervention [as] a way of learning [that can be used] as a basis for intervening in the world’. He goes on to describe that this process must ‘afford [the individual] the opportunity to read, write and learn from a position of agency’. Doing so enables the attainment of information together with the creation and consolidation of new knowledge based upon ‘the conditions of their current lives’. However, with the acquisition and consolidation of new knowledge we suggest that there was also skills development. Therefore, adopting Giroux’s [33] position, a theme related to the acquisition and establishment of knowledge and skills development emerged from the data. For this second theme, the behavioral descriptors for knowledge were (i) ‘information providing’ and (ii) ‘knowledge encoding’. These emerging themes were supported by Wolfe et al.’s [20] theory of health learning capacity, which has two main constructs that are associated with health cognitions and psychosocial skills (e.g., communication). The workshops allowed for knowledge skills development by enabling the participants to construct new knowledge through their reasoning (i.e., health cognitions) and verbal abilities (i.e., communications). 

Theme 2 ‘constructing knowledge’: using their life experiences, the young people reflected upon and encoded the content of the workshops. Verbally expressing their ideas on sensitive issues affecting them and listening to others, the young people increased critical thinking and improved their communication and conveyed their new knowledge to others. The young people, therefore, spoke of how the information was accessible and meaningful to them, encouraging new habits. The following comments are illustrative: ‘I learnt about different foods’ [workshop on health eating] (YP 4); ‘I learnt about levels of sugar content in different drinks’ (YP 10) [workshop on oral health]; ‘I liked everything because I learnt a lot’ (YP 1); ‘It was really helpful’ (YP 3); ‘the best part of the workshop was to know how to overcome drug abuse and addiction’ [workshop on substance misuse] (YP 1); and, ‘I understand more about mental health’ (YP 5) [workshop on mental health]. 

The inclusion of their own experiences, during constructing knowledge, permitted an encoding of the health information provided leading to an increased health awareness. The quote from a young man experiencing homelessness highlights this:

‘I really enjoyed it because you talked about the use of cigarettes as well, the use of drugs, and that was really helpful for me to understand the effects that I will get after using drugs. So in that way it helped me to stay far from taking drugs. Really helpful to my health’. (YP 1)

Integrating the information into dynamic group activities thus afforded a platform for discussion (‘I could talk about normal life, issues related to me’—YP 11) and enabled the construction of useful new personal knowledge, (as above), through the structuring of the information provided (‘knowledge encoding’). Freeman [21] has postulated that it is within the context of people’s social worlds that individuals are able to ‘manipulate, encode and transform’ health information into a useable form. The sharing of health information as part of knowledge encoding between young people and their service providers provided an opportunity for the converting of new information into an understandable and useable form. This was achieved by encouraging dialogue and the using of imaginative and diverse artistic learning activities to enable participants to express their feelings and opinions beyond the spoken word, therefore improving the psychosocial skill of communication. This was most apparent during the Resilience workshop that used a card game to build emotional strength. The young people had to choose and comment on quotes from famous people for transforming the thinking of their times:

“The session that I liked the most was the card games, yeah, that’s true. There were quotes we had to choose, and I really liked the quote from Nelson Mandela saying about failures and stuff. The one on the importance of rising every time we fall. Yeah, it was really good to think about this”. [the workshop on Resilience] (YP 6).

‘I think the use of educational games was great. I particularly think the last session that we had on resilience was one of the best discussions I’ve had doing a group here. I mean the whole time I’ve been working here. Because it’s very difficult to get young people to open up in that way and talk more widely about their bigger life goals and what they find scary. And I think the fact that they were happy to do so was a testament to the relationship that you’ve managed to build with them and also the resources available’. (SM 3)

Information providing and knowledge encoding, as descriptors for constructing knowledge, acted as precursors for ‘developing skills’ and behavior change. Skills development appeared to be associated with active learning, consensus building, and agreed action plans for future behavior change. Consensus building was an important dimension of knowing and it was used to combine group knowledge, for instance, for oral health-related behaviors. 

“I was working with a young lad who came to a couple of the workshops, he had a workshop about dental health and a discussion about sugar and diet and stuff. And I know that he doesn’t drink those energy drinks anymore, which is fantastic. Because he was drinking maybe one or two cans a day. And when you realize how much sugar was in them, because we had that visual exercise of how much sugar is in things, and I think that actually struck a chord with a few people, so I think that was really beneficial”. (SM 5)

Therefore, the merging of old and new knowledge, within the group, supported new knowledge construction and its conversion into action plans for lifestyle change, as detailed in Table 3.

Theme 2 ‘knowledge conveying’: the second behavior associated with the developing skills. Many of the young people suggested that they believed that they could tell, speak, or spread their new health knowledge among their friends and family:

“Basically, the main thing I’ve learned from the workshops, from being homeless and my journey is to respect and listen to other people because there are people who know more than you and you don’t know everything. Take things that people say and take it on board, and everything’s a learning curve, you learn things all the time... And I’d recommend that to anybody else who is homeless, just listen to other people, take on board what they’ve got to say, and accept the help that’s around you like the group activity [the workshops]”. (YP 3)

It may be suggested that a skill outcome of the workshop program was the strengthening of the young people’s social interacting by equipping them with confidence to speak to others about health-related issues. This confirms the proposition that the workshop’s pedagogical structure had provided the means by which young people could encode, transform, incorporate, and also disseminate new workshop information into a useful working knowledge language. For Wolf et al. [20], this would be evidence of improved health learning capacity: for Giroux’s [33] it represents, ‘a basis for intervening in the world’: 

“I think it’s a very good thing [the workshops] because when you provide information about your own health, to people, it helps in their lives, and in that way you might help the same people who have got that information to tell other people... and these other people may someday come to get this information”. (YP 6)

The participating staff members noted changes in the culture of the NGO following the program. They spoke of how the pedagogical workshops had provided them with new knowledge and increased their own awareness of the necessity of creating a supportive environment with and for young people: 

“So I think the more we talk about people’s health and how they can do things to help themselves, and more enabling stuff to give themselves the power into doing their own decision making in a positive way, then I think that should be strongly encouraged”. (SM 1)

By joint working and the inclusion of people’s rights and citizenship, as essential elements in health promotion interventions, they stated that the pedagogical workshop program had provided a strategy in the NGO to support young people who were experiencing homelessness. Moreover, there was a common understanding that all processes of change, especially those that are related with health improvement of people experiencing homelessness, were challenging for practitioners and service providers. 

“I think sometimes health behaviors are some of the most difficult to change quite often I do come across young people who have got some pretty unhealthy habits in terms of smoking, eating, sleeping, that sort of thing but they do seem quite resistant to change”. (SM 2) 

## 4. Discussion

The aim of the work presented here was to use Freire’s critical consciousness as an educative tool, to co-design, implement, and evaluate a series of pedagogical workshops to strengthen social interacting, critical consciousness, and to construct new knowledge, with, and for, homeless young people and their service providers. The findings showed the importance of incorporating this approach. The results intimated that shifts in self-regard and behavior of the young participants had occurred. Wider health topics and sensitive issues of their homelessness journeys were explored through their own lens in face of marginalization and exclusion. Regarding the delivery of the pedagogical workshop program, there was a positive and common feeling from the participants about key elements that were prioritized: the offer of a welcoming, safe, creative, and pleasant space to speak, share, and listen to other’s narratives, and perceptions around each topic explored during the workshops. In this atmosphere, the participants were able to discuss issues that caused them concern and through the process of critical reflexion, collective construction of new knowledge and behaviors were achieved. The underpinning basis of the workshops was the adoption of a CRFA that connected oral health with other diverse topics. For example, the workshop on diet and nutrition was related to the consumption of sugars and hence oral health; the workshop on stigma brought up concerns about appearance of their teeth linked with social interaction and seeking employment opportunities, besides judgments made about their drug use and so reflected the concept of inclusion oral health. The importance of including the mental health workshop allowed dental anxiety, oral health-related quality of life and depression to be raised and explored by the participants. This was relevant, as previous research had shown that decayed and missing teeth were predictive of depression in homelessness [34]. Therefore, this workshop intervention was central to, among other aspects, the promotion of oral health, and health through increasing oral health literacy [20], to build trust among their peers and collective engaging with the service provider. 

While this was realized using a process of mutual learning and the construction of new relationships, we suggest other factors were instrumental in establishing a safe and trustful place for spontaneous social interacting. A non-judgmental listening was crucial to involve vulnerable groups in discussions that affect their own health and wellbeing. We proposed that the safe place was created by ‘locating [the participants] in the condition of their current lives’ [33] together with increasing their awareness that they were not alone. The establishment of a place of safety to discuss sensitive issues, therefore, would appear to mimic a therapeutic space, which Bell et al. [35] consider to be of significance to ‘maintain and promote health and wellbeing for different individuals and groups at different times’. It was within this therapeutic space that the assessment, re-assessment and reflection of past experiences, which had raised the levels of stigmatization and humiliation, were at last reconsidered, thereby reducing feelings of shame and inappropriateness [25,26]. We propose that critical consciousness as an educative tool conjured a therapeutic space for spontaneous social interacting—an ‘empathetic encounter’ to strengthen social engagement and consolidate new knowledge. In other words, the level of critical self-consciousness that was achieved by the young people had resulted in a better awareness of their relationship with their health and their wider attitudes as citizens.

In addition, some support for behavioral change was found. For instance, at the end of the workshop program, new health habits were noted in the participants through the post-questionnaire’s evaluation and practitioners’ feedback. Reports of speaking about, and sharing, difficult health and life experiences; critical reflexion on sensitive issues; working with others to form agreed action plans for health; together with the desire to act with peers and family to disseminate health messages, indicated to us that a behavioral shift had occurred. The young people became the ‘problem-posers’ or the questioners and as a consequence became involved in a dialogue about their oral health and wider health issues. The significance of this shift, while being related to their acquisition of new knowledge and improved self-regard, also aided young people to assist in changing the culture of the NGO. The main objective of the workshops was to offer to the participants a space for critical reflection on themes present in their lives in a welcoming, dynamic, and creative atmosphere that can encourage new actions and behaviors. The whole process follows the critical-reflexive line that was proposed by Freire, based on the integration of the previous knowledge of the young people with the appropriation of new and contextualized knowledge and practices.

Critical reflexion and confrontation of current life circumstances (in this case being young and homeless), as part of this critical consciousness proposed by Freire, allowed for participants to reflect upon their status, self-stigmatization, their responsibilities, and their roles in terms of social transformation. Young people and practitioners, thus, developed an increased understanding of the youth homelessness life context; how past health choices and experiences affects their current life and how this new knowledge acquisition strengthens future social change.

Limitations:

This work explored the use of a co-designed pedagogical workshop program to promote the acquisition of new knowledge and social interaction in a group of young people living in supported accommodation and attending activities that were provided by a NGO for homelessness. 

A relatively small number of young homeless individuals took part and this calls into question the generalizability of the findings and conclusions to others experiencing social exclusion. However, while acknowledging the small sample size, this was a purposive and non-probability sample of a group of vulnerable homeless youth, who found it difficult to interact and engage with health and social care services and communities. They had, and were, experiencing social exclusion in diverse levels. The similarity of their comments and the merging themes suggested that saturation regarding their opinions and views of the workshops had been achieved and thus provided a form of validity to the findings. In addition, we were not able to comment on the longer-term behavior change at this stage of the research. Therefore, the findings of this qualitative evaluation provide a platform to allow future work on young vulnerable people, exploring co-designed interventions with this group to improve their engagement with services, health literacy, and enable social inclusion. 

## 5. Conclusions

In conclusion, we propose that Freire’s educational approach provides a useful framework to promote health and oral health with young people experiencing homelessness. It allowed the young people to be included in the co-design of the research intervention and enabled their active participation, building trust, and interaction with others within the NGO setting. In this process, opportunities to identify and to voice their health needs were explored, and their health learning capacity to make conscious, positive, and healthy choices was improved. Therefore, while accepting that there is a need for further research development in other NGO settings, this work suggests that the use of critical consciousness supports young socially excluded people’s construction of new knowledge, health literacy, and strengthens their social interacting among their peers and engagement with services providers. 

Other elements of this debate are related to the difficulties that are experienced with regard to behavior change among youths experiencing homelessness. We suggest that the development of agreed action plans during the workshops and subsequent noted behavior change were associated with the workshop program ‘being located in the conditions of the current lives’ of the service users and providers and the generation of a therapeutic space. It is expected that educational and research activities, such as the workshops, using Freire’s theory, can contribute to listening to the voices of vulnerable and marginalized youths, encouraging them to adopt healthy life choices and also support them to achieve a critical consciousness and participatory citizenship.

## Figures and Tables

**Table 1:** Pedagogical workshop development.

Developmental Phase	Procedure	Outcomes
Phase 1	The selection of a key NGO supporting youth homeless in Scotland through a preliminary mapping of homelessness services and organizations. A series of meetings between the PI with staff members of the NGO and with youth homeless living in temporary accommodations. Identification of the main topics to be covered through the workshops as agreed by all participants. Co-design and delivery of the workshops.	An initial package of four workshops planned as requested by staff members and the young people, delivered and evaluated. Topics: [i] Oral health, [ii] Mental health, [iii] Education & the future, [iv] Stigma Planning of the workshops involved a multidisciplinary collaboration between the PI with one NHS Board and one NGO to provide expert knowledge on the evidence-base and current guidance on oral health and mental health. Evaluation by direct observation on participants’ feedback and group discussion during the workshops, and post-workshop questionnaires.
Phase 2	Following the same participative approach, the feedback received following Phase 1 was positive, and the young people requested further workshops. Meetings with young people were convened to discuss openly and using critical consciousness, to identify the additional workshop topics regarding the workshop package 2. Co-design and delivery of the workshops.	A second package of workshops was planned, delivered and evaluated following the same procedure as in Phase 1. Topics requested by the young people: [i] Homeless trajectory, [ii] Substance misuse, [iii] Resilience, [iv] Healthy eating Planning of the workshops again involved a multidisciplinary collaboration between the PI with one NHS Board and one NGO to provide expert knowledge on the evidence-base and current guidance on substance use, resilience, and healthy eating.
Phase 3	Semi-structured interviews with all participants to explore key issues raised during the workshops including perceptions of the efficacy and appropriateness of using pedagogical workshops to explore the life experiences, views and opinions of the young people	Identification of key issues and perceptions of efficacy and appropriateness of workshops.

**Table 2:** Content and implementation of co-designed workshops 1–8.

Workshop 1. Oral health	Exploration of group perceptions of oral health, fears and barriers to accessing dental treatment emerged. A spontaneous discussion of the psychosocial effects of poor oral health on self-esteem, social interaction, and employability occurred. Common and divergent experiences were then identified and built into collective strategies to achieve good oral health as a way to improve other aspects of life.
Workshop 2. Mental health	[Part 1] Exploration of group perceptions of wellbeing and mental health; health information on different mental health problems and their causes; treatments available; role of practitioners, friends and family to tackle mental health problem. [Part 2] Sensitive discussions of relationship breakdown as a cause of mental health problems among young people; sharing of life experiences with family members and/or partners; the group discussed how to improve communication; how to manage conflicts, differing beliefs and world views; consensus of how these factors can affected their well-being and mental health.
Workshop 3. Education & the future	[Part 1] The participants were asked to identify and discuss different levels of knowledge in their lives built from both formal education (e.g., courses, college or university) and life experience; the recognition that both types of learning are important and serve the context required. [Part 2] Discussion of aspirations for the future. To visualise this future the participants built a collage to express a life project involving and promoting their health and wellbeing. [Part 3] Identification of feelings coming with the life planning exercise and an exploration of how they would make healthier choices for a better future.
Workshop 4. Stigma	The aim of this workshop was to continue to humanise the gaze and to enable participants to discuss the meaning and process of the construction of stigma against groups in society and especially youth homelessness. Using favelas in Brazil and their youth residents as an example of a stigmatized group, the participants felt comfortable to bring their own experiences of bias and stigma and the agreed strategies they would use to deal with discrimination. The participants were invited to create a campaign against prejudice, stigma and discrimination towards homelessness.
Workshop 5. Homeless trajectory	Discussions around participants’ definitions of being young and their homelessness journey; exploration of the positive/negative aspects of this period of their lives; identifying routes that lead to their homelessness. The group was invited to engage individually and/or as a group in activities to produce a consensus outcome using diverse and creative ways of expression to translate this knowledge into an action plan for their lives.
Workshop 6. Substance misuse	Increased participants’ awareness and knowledge of substance misuse; focussing on the increased use of New Psychoactive Substances (NPS), by young people; exploration of their reasons for becoming involved in drug use; participants’ consensus of how to deal with drug use and how to overcome involvement.
Workshop 7. Resilience	Discussion of the meaning of resilience as the capacity to adapt and overcome risk and adversity; exploration of how they may become more resilient; learning of life skills and strategies to build their strength when going through a difficult time. Through the identification of positive thoughts that lead to their social change and negative thoughts that hold them back, the participants worked on the construction of their resilience, their life goals for future planning and the different steps necessary to achieve these goals (an action plan).
Workshop 8. Health eating	Discussion of the role of food in people’s daily lives beyond the survival aspect; raising awareness and understanding of food as a way to [1] encourage social interaction, [2] break cultural and social barriers, [3] engage with people, [4] increase health literacy and [5] promote wellbeing. The participants reflected upon health eating and the effect of their position and social inequalities as the main reason why people living in poverty do not eat healthy food. This provided the basis for a co-construction of knowledge and an action plan for healthy eating.

**Table 3:** Consensus building and joint action plans for oral health (OH) behavior change following OH workshop.

Quotes Related with the Workshop on Oral Health	
[1]	(I am) brushing my teeth differently (as a result of the workshop on how to brush teeth with fluoride toothpaste);
[2]	(I am) using mouthwash at different times [as a result of information about not using a mouthwash immediately after brushing teeth;
[3]	(I will) not use water when brushing my teeth as it’ll wash away the concentrated fluoride in the remaining toothpaste’ (as a result of the knowledge gained at the workshop);
[4]	‘I now use a pea size of my toothpaste’ (following the information given at the OH workshop when the facilitator asked a participant to show the right amount of toothpaste that should be used to brush the teeth and the amount showed was far away more than the recommended by NHS boards);
[5]	I am using a straw to drink any juice’ (as result of the workshop on OH and diet that revealed the high amount of sugar present in the carbonated drinks consumed by the young people. The information caused a lot of surprise among the participants. The workshop provided advice on how to minimize the effects of these drinks with simple tips such as using a straw;
